# Caring Process in Hematopoietic Stem Cell Transplantation: A Grounded Theory Study

**Published:** 2019-04-01

**Authors:** Leila Sayadi, Vahid Zamanzadeh, Leila Valizadeh, Fariba Taleghani

**Affiliations:** 1 Nursing and Midwifery Care Research Center, School of Nursing and Midwifery, Tehran University of Medical Sciences, Tehran, Iran; 2 Hematology-Oncology and Stem Cell Transplantation Research Center, Shariati Hospital, Tehran University of Medical Sciences, Tehran, Iran; 3 Faculty of Nursing and Midwifery, Tabriz University of Medical Sciences, Tabriz, Iran; 4 Nursing and Midwifery Care Research Center, School of Nursing and Midwifery, Isfahan University of Medical Sciences, Isfahan, Iran

**Keywords:** Hematopoietic stem cell transplantation, Caring, Grounded theory, Supporting

## Abstract

**Background:** Caring is one of the main concepts in nursing and its modes of delivery in different diseases have been widely studied. Hematopoietic Stem Cell Transplantation (HSCT) is a novel, complex, and time-consuming clinical intervention which is applied as a final medical choice in several life-threatening diseases. The aim of the current study was to explore the process of caring for patients undergoing HSCT.

**Materials and Methods:** In this article, we present a qualitative research study conducted between 2011 and 2013 in accordance with the procedures of grounded theory methodology. Data were gathered by interviewing and observing health professionals involved in HSCT process, as well as patients and their families. The study participants consisted of 18 HSCT nurses, 2 physicians, 12 patients, and 7 members of patients’ families. The initial sampling in the study was purposeful, followed by theoretical sampling. Data were analyzed using the Corbin & Strauss (2008) method.

**Results:** Four main categories, reflecting 13 sub-categories, were emerged by analyzing the data: struggling of patients between life and death, trying to reduce the chance of patient’s death, enforcing patients’ spirit and caring achievements. The core variable of study, defined as “supporting patients to go through the HSCT process successfully”, represented the nature and efficiency of care delivered to HSCT patients in the study setting.

**Conclusion: **HSCT patients enter the caring process in the context of life-and-death limbo. The caring strategy in HSCT patients is aimed at trying to reduce the chance of the patient’s death, as well as enforcing patients’ spirit. The HSCT process affects all areas involved in various ways and has some outcomes. The findings and the theoretical conclusions of this study are potentially valuable in improving nursing practice, designing of educational programs and setting of caring policies.

## Introduction

 Caring has been considered as an art^1^, goal, and mission^2^, as well as the essence and central focus of nursing^4^^,^^3^, its metaparadig^5^^,^^3^, and equivalent to the profession itself^2^. Caring is also the axial concept of nursing research^6^, and a multitude of studies have been conducted in the fields of caring and caring models in nursing^8^^,^^7^, meta-synthesis of care^9^, and concept analysis of care^10^. Caring; however, remains a vague and debated concept in nursing, making the necessity of further research and theorizing on nursing care a scientific inevitability^12^^,^^11^.

Studying the delivery of care to different groups of patients can enlighten the concept of caring^13^. Several studies have been performed from the perspective of working in intensive care units^14^ cancer^15^, psychiatric^12^, and burn wards^16^, as well as from the points of views of the caregivers to dying patients^       17 ^ and patients with advance illnesses, suffering from breathlessness at home^18^, and also from the standpoint of cancer patients^19^. Other studies have probed the experiences of nurses with delivering palliative care to cancer patients^20^, cancer patients in non-specialist wards^21^, and chemotherapy patients^22^. However, we were unable to find any studies addressing the concept of care for HSCT patients.

HSCT is a complex, time-consuming, and high-cost medical intervention ^23^^, ^^24^ for several otherwise fatal diseases such as hematological malignancies, solid tumors, aplastic anemia, autoimmune disorders, congenital immunodeficiency syndromes, and metabolic disorders^25^.

HSCT experience is one of the most stressful of all treatments^26^. The patients face physical incapacities, as well as social and psychological disturbances^27^. These patients’ complications are even more severe than those of cancer patients^28^          since HSCT is a high-risk treatment with potentially life-threatening complications and no definite perfect outcome^23^^,^^27^. 

Global use of HSCT is rapidly increasing despite its complexity and high cost^24^, and currently this mode of treatment is conducted in over 500 medical centers located in 50 countries^29^. Statistics suggest that more 50,000 patients are treated with this procedure annually across the globe^25^. Iran with the incidence of 1400 diseases requiring HSCT per year is one of the pioneer countries to adopt this mode of treatment^30^^,^^31^. Since the establishment of the first HSCT center in Iran in 1990 up to 2011, 3237 patients have been treated with this medical intervention^32^. 

HSCT is a relatively new mode of treatment and there still exist unanswered questions about care delivery to HSCT patients, necessitating further research^       33 ^. Delivery of care to HSCT patients is a multi-dimensional challenge, open to various interpretations. That is why studying the caring for HSCT patients and improving the quality of this care has been considered a priority^34^.

The majority of nursing or multi-disciplinary researches conducted worldwide on HSCT have quantitatively^35^ or qualitatively^36^^-^^38^ addressed many specific aspects of this intervention such as managing drug-induced toxicities, caring for patients with physical complications, devising guidelines on providing psychiatric care, HSCT patients’ quality of life, and HSCT patients’ perspectives and problems     ^26^^,^^28^^,^^39^^,^^40^ . However, a theory of caring for HSCT patients, which is an apparent necessity of the nursing profession^10^ is yet to be developed.

Taking into account the fact that caring patterns and procedures are deeply rooted in local cultural settings and backgrounds^41^ and bearing in mind the necessity of studying the concept of care in Iran^42^, conducting a qualitative study about the process of providing care to HSCT patients is considered to be suitable for practical use. In this respect, the present study aimed to explore the process of care for HSCT patients and provide a caring theory, using the grounded theory’s qualitative approach. Results of this study can offer rich description of caring for HSCT patients.

## MATERIALS AND METHODS

 A qualitative, grounded theory approach was used to study the process of caring in HSCT. Grounded theory is the most appropriate method of research when there is limited data, and little is known in an area. In the grounded theory, concepts and theory are derived from the data^43^. Grounded theory is based on symbolic interaction, and social symbolic interaction is at the heart of caring process^   44 ^. Caring is an interactive process^   45 ^, cohesive, and context- specific interpersonal process^9^.

Grand theory is used to bring a broad view to nursing practice and research. Nursing is a complicated topic which needs development of theories related to its practice^46^. This approach helps in explaining the concepts generated from data gathered from the participants in research, interpreting the reality, making clear the different meanings of phenomenon, enhancing the insight and understanding, and finally providing a guide for action^43^.

**Setting**

The present study was performed in the main HSCT center affiliated with Tehran University of Medical Sciences between 2011 and 2012. This center consisted of three adult HSCT wards, one pediatric HSCT ward; two hematology and oncology wards as well as one post-HSCT ward. 

In total, 67 nurses with a bachelor's degree in nursing were working in these wards. The most common transplanted disorders were acute myelogenous leukemia, thalassemia major and acute lymphoblastic leukemia. The mean age of patients was 23 years, and the median age ranged from 4 months to 71 years^47^.

**Participants**

In grounded theory, sampling begins by recruiting participants who will be able to provide the best data to answer the research question^   43 ^. Participants included 18 HSCT nurses, 12 HSCT patients, 2 HSCT physicians and 7 HSCT patients’ family members. The characteristics of the participants are shown in Table 1. 

**Table 1:** Participants characteristics

	**Participants**			
	**Nurses**	**Patients**	**Physicions**	**Family Members**
Numbers	18	2	12	7
Mean of age(range)/ Years	37.4 (25-53)	43 (40-45)	35.8 (20- 61)	40 (24-55)
Sex				
Female	18	1	6	5
Male		1	6	2
HSCT work experience Mean(range)	9(1-21)	3(1-5)		
HSCT ward				
Adult HSCT	10			
Pediatric HSCT	2			
Post HSCT	6			
Patients based on type of disease				
Leukemia			7	
Aplastic anemia			2	
Hodgkin's lymphoma			2	
Non-Hodgkin Lymphoma			1	

Inclusion criteria were the following: (1) receiving at least one-year experience of HSCT caring; (2) having direct HSCT caring in 6 last months (for nurses); (3) having at least one- year experience of HSCT treatment (for physicians); (4) being *≥ 18 years* and (5) having not physically and mentally weakness (for patients). Family members who were directly responsible for caring the HSCT patients were also entered the study. Both patients and family members had to speak Farsi fluently.

In this study, we used the maximum variation sampling strategy^43^. Since the researcher had many years of experience in the field, first, purposeful sampling was used, and initial participants were selected from nurses with higher degree of cooperation, experience and information. Then, after analyzing the primary data, targeted sampling was changed to theoretical sampling, and participants were selected based on obtained information and results of the primary analysis. At this point, properties and dimensions of the concepts were developed, and the relationship between concepts was identified^43^. Sampling continued until saturation and the development of theory.

**Data collection**

One of the characteristics of qualitative research is that multiple sources of data can be used^43^. In the present study, both interview and observation were used. 

**Interviews**

Although the interview guides evolved during the process of the study^43^, formal semi-structured interviews were conducted. Initial interviews asked participants broad questions about caring and perceptions of caring. For example: How do you take care of HSCT patients? What do you feel in caring for HSCT patients? 

Interview questions were later refined to include more specific questions about concepts which emerged from earlier analyses. As data collection progressed, interview questions were refined in accordance with the concept of theoretical sampling and the interaction of data collection and analysis^43^. 

At first, the aim of the study, the method of interview, the need to record the interview were explained to all participants, and if they gave their consent, the interview was performed. Participants were also informed that their *personal *information would not be disclosed. Some participants were interviewed more than once. A total of 46 interviews were conducted between 30 to 120 minutes in length.

Some informal interviews were also conducted with nurses during observations which were not planed in advance. These informal interviews were performed during casual encounter with nurses as a participant observation^48^^,^^49^.

**Observation**

Observation is an essential method for gathering data about the behavior of participants in special circumstances and its combination with interviews is beneficial^43^. The data from observations includes detailed descriptions of people’s activities, behaviors, actions and the full range of interpersonal interactions and organizational processes^46^.

The presence in the research environment and observation of related activities were all coordinated with authorities. The researcher acted as an absolute observer, and all observations were performed in an unstructured manner, which captured the social setting in which people functioned^50^.

Environment and organizational qualities of HSCT wards, activities, behaviors and interactions between nurses, physicians, patients, and their families as well as all events happening in the wards, were the subject of the researcher observations. Besides, the researcher took notes during and after the end of each observation and analyzed those observations. In total, 18 observations were performed from participating wards in a period of 78 hours.

Field notes were used to document the activities in the ward as well as researcher’s interpretation of these activities. The researcher conducted the writing of field notes after each interview and during the presence in the research environment. In this study, the observational notes included an objective description of events, conversations, dialogues, information about the context and the field environment^51^.

When the researcher concluded that data saturation was achieved and no new data was acquired, categories and their relationships were determined and the data collection was stopped.

**Data Analysis**

The method of data analysis used in this study was Corbin and Strauss (2008) approach. The researcher analyzed the data through a step-by-step process: analyzing data for concepts, elaborating the analysis, analysis of data for context, bringing process into the analysis and integration of categories^43^. Each recorded interview was transferred to MAXQDA software with observations and field notes. Data analysis began as soon as the first data were collected to ensure interplay between analysis and data collection ^43^.

Data analysis was performed with open and axial coding synchronization and began after the first interview^43^. The primary classification was performed based on extracted codes and codes were categorized based on their differences and similarities, and then primary concepts were formed. Encoding each of the interviews and observations was conducted simultaneously after comparison with previous data, which is the most important strategy to elaborating the analysis^43^. Concepts and categories related to context were then explored. At this stage, the researcher tried to explore the conditions that led to events in which the study participants answered with actions, interactions and feelings^43^. Analysis at this stage showed that care for HSCT patients is conducted while the patients are struggling between life and death.

What guided the next step were what actions / interactions/ emotions occurred in response to this limbo between life and death and what the consequences were. Concepts and memos of the analysis were reviewed to discover the process behind them.

In the entire process of collecting and analyzing data as well as developing a theory, memos^   43 ^ were used. The researcher would write opinions and thoughts in interaction with the data and would make a note of any decision which was made in the process of data analysis.

These notes would make an idea of the process in the researcher’s mind. An example of these memos which helped in theory formation was as follow: “There is a multitude of problems ahead of HSCT patients and they are at the beginning of a dangerous road which they should travel. It seems that all nurses are putting a lot of effort in helping patients to pass through this dangerous road.” 

After this stage, the researcher looked for the core categories and abstract words which would explain other categories. To achieve the core categories, the researcher began to write the narrative of the whole study. The main concepts of the study, relationship between them, memos and diagrams were studied several times, and core categories were identified based on these elements.

**Rigor**

To increase credibility, the researcher had a prolonged engagement, and the collection of data and its analysis took more than one year to complete. The researcher used a combination (triangulation) of observation and interviews to gather data from nurses, physicians, patients and their family. To increase the reliability of findings, the researcher used a checking process with the participants: the concepts and categories derived from the data were checked with some of the participating nurses for their accuracy. The researcher also used a peer checking process in which the steps of data analysis would be relayed to authors, and the reliability of data and its analysis would be judged. For dependability, all steps of the research were clearly discussed; the authors approved the process of the study and were aware of the entire process of data analysis and the formation of theory from the data. To increase the transferability, a clear explanation for participants, field research, the method of data gathering and the formation of theories are provided in this study. The researcher also chose the participants from different age groups, work histories, HSCT duration, and ward hospitalization to increase the generalizability of data.

**Ethical considerations**

The study was approved by Ethics Committee of Tabriz University of Medical Sciences and the Hematology-Oncology and Stem Cell Transplantation Research Center. Participation was voluntary in the study and the participants were free to withdraw from the study at any stage. 

**Findings**

**Figure 1:**
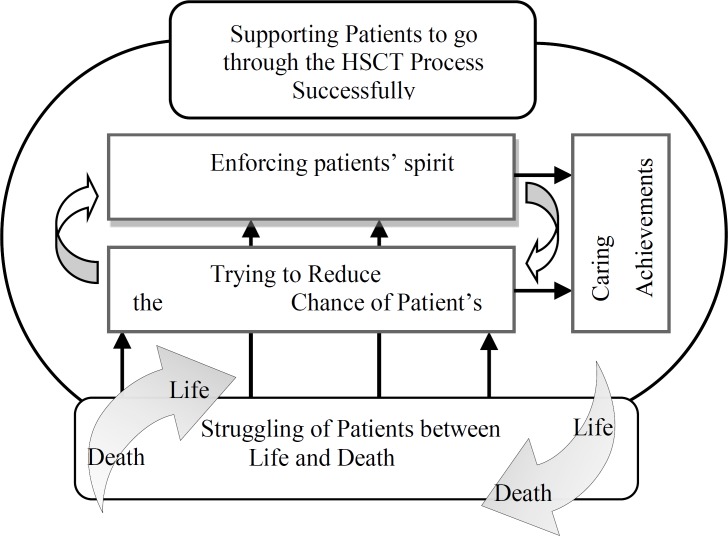
Integration of main categories around the Supporting Patients to go through the HSCT Process Successfully

Data analysis showed four categories which summarize the caring process in HSCT: A) struggling between life and death, B) trying to reduce the chance of patients' death, C) enforcing patients’ spirit and D) caring achievements (Table 2). The core variable of the study was "supporting patients to go through the HSCT successfully" and all other categories were around this central concept (Figure 1). 

**Table 2:** Main Categories and sub categories

**Main** ** Categories **	**Sub ** **Categories**
Struggle of Patients between Life and Death	Patients under the shadow of death
	Living of patients with uncertainty
	Deep involvement with patient’ problems
Trying to Reduce the Chance of Patients’ Death	Prevention
	Monitoring
	Management of dangers and side effects
	Follow up
	Continuing education
Enforcing Patients’ Spirit	Consolation
	Instilling hope
	Fading out the hard realities
Caring Achievements	Patients passing the HSCT
	Emotional Labour

The findings indicated that the main psychosocial problem in the process of patient care is the struggle of patients between life and death. HSCT is a time -consuming process and patients are at risk of various dangers before and after HSCT.

*“There is a probability for HSCT rejection for years, so we cannot say the patients will be cured in a determined time frame; there is no end to their disease.”(Nurse 1)*

Therefore, there is always the risk of death for these patients. In other words, nurses are faced with patients who are under the shadow of death. Patients have many difficulties before HSCT, and other challenging problems happen during hospitalization. Nurses are facing patients who are desperate, and HSCT is their last hope and at the same time should endure a multitude of hardships which affects their situations:

*“When they come for HSCT, it means other treatments have not worked, and HSCT is their last chance.” (Nurse 14)*

In response to this situation, nurses try to reduce the patients' risk of death and control the other risks. They try to prevent bad outcomes by monitoring the patients for signs of infection, bleeding and complications of HSCT.

*“We are working ahead of schedule.......trying to give the best care.........trying not to put patients’ life in danger........sometimes there is no time for physician to come and give an order....we cannot wait for them to come and order.”(Nurse 16)*

Patients are under constant surveillance, necessary tests are performed and evaluated and drug complications are examined.

*“Patients should be monitored constantly, and in every working shift, we continuously check them to make sure they have no problem.” (Nurse 5)*

However, many HSCT problems and drug complications may occur in patients which nurses manage them appropriately using existing protocols. These complications are followed even after patients are discharged from hospital: 

*“Patients' care within the ward environment is highly significant, but professional support will be continued after hospital discharge. The patients will be followed-up to* *prevent and treat complications and to assess engraftment and disease status.”* *(Nurse 5)*

In parallel with all these efforts, to reduce the risk of death, the education of patients and family starts before hospitalization and continues after discharge from hospital: 

*“Nurses hold classes for patients and their family, the nurses give them basic information about HSCT and its stages, chemotherapy, autologous and allogeneic HSCT, necessary procedures like CV line, etc. They also provide them with information concerning the ward rules such as the visiting hours and isolated rooms.” (Notes of researcher observation)*

*“In the first days, when patients are hospitalized we teach them how to keep clean their room, how to take a bath, how to control I&O; we give them a measure and based on their education explain to them how to measure or what is the volume of glass.”(Nurse 3)*

*“Nurses told me how to keep the house clean after discharge.....how to keep the visitors* t*o the minimum number possible...they also provided instructions about home care.”(Patient family member 2)*

*Patients*' *spiritual* outlook is important to handle health difficulties after HSCT. Helping patients *spiritually* is the primary aspect of care for these patients.

*“Patients with a strong fighting spirit may have a better outlook. We have seen that patients with spiritual crisis usually encounter treatment problems.” (Nurse 2)*

*“* *Good spiritual health is an important factor for patients* *, and we have seen that patients with a high spirituality level are discharged from hospital more quickly than sad and hopeless patients.... depressed patients make their problems worse.”(Nurse 1)*

Consoling, instilling hope and fading out the bitter realities are the steps taken to enforce the patient's spirit. Using these methods help patients to better pass through HSCT difficulties:

*“I used to tell them: this is one of the stages of your life; you should go through it.”(Nurse 2)*

*“I tell patients imagine you are cured.* *When you think positive, good things happen.” (Nurse 9)*

Over time, patients accept their situation little by little.

*“It is all about patience. I had patience; it is truly thanks to God that I am in this stage. It is destiny… my life destiny. I will cope these tough times, hard days. I thank God.”(Patient 2)*

*“I am keeping myself up...I have coped with various problems caused by treatment procedure.” (Patient 12)*

Some patients have a successful HSCT, and live for a long time after it:

*“We had a kid who received HSCT and now goes to school; we had single patients who are now married or young patients who go to university now.”(Nurse 4)*

*“We had an ALL patient who received HSCT in 1999, went to India and studied dentistry, and then decided to enter specialty field (Nurse 7)*

 But, there are also patients who expire because of the numerous complications: 

*“Many patients expire.”(Nurse 5)*

On the other hand, there is a kind of friendship between the nurse and patient during the care process:

*“Our patients become very dependent on us, this is a two-way bond.” (Nurse 2)*

*“Our friendship has continued even 10 years after my HSCT. My wife is in contact with them, and they are friends. One of them brought us a gift when we had our first child, I keep sending greeting cards for particular occasions such as New Year and Nurses' days.”(Patient 10)*

This close relationship sometimes have negative or positive emotional labor for nurses:

*“I am in a negative emotional state since I came here.” (Nurse 10)*

*“I feel I am a better person now. Nothing is important anymore………….I have become quieter, even kinder and more generous.” (Nurse 13)*

Data shows that nurses try to answer their main concerns about “struggling of patients between life and death” in the course of HSCT care by trying to reduce the chance of death among patients and enforce the patients' spirit by doing activities which have supportive qualities:

*“I try to support my patients in any possible way.”(Nurse 10)*

*“Caring means supporting of patients; we try to be supportive.”(Nurse 2)*

*“We support our patients. Spiritual support is essential for these patients; it might even be more important than other things.” (Nurse 1)*

*“The Support of patients is very important.”(Nurse 9)*

*“What we do for these patients is providing supportive care: psychotherapy and physical therapy.” (Nurse 14)*

This support not only reduces the chance of death among patients but also promote the good spirit among them. Nurses try to bring back the good spirit among patients which might be lost in the process of treatment and HSCT. Nurses emphasized on the supportive portion of their care, while their main focus was on successful passage of patients through HSCT procedure. In other words, supportive actions are nurses’ behavior during the care for HSCT patients to achieve successful treatment. So, the main variable is considered to be supporting patients to go through the HSCT successfully.

Supporting patients to go through the HSCT process successfully was a variable with high emphasis in the data, and was observed numerous times in the course of the study. The supportive care of patients had different physical, psychological, spiritual and socioeconomic aspects, and was performed while patients were fighting for their life. 

Nurses were trying to reduce the dangers of treatment for patients, and at the same time, uplift the patient’ spirit to pass this hard passage of their lives. The achievement of this supportive care was the passage of patients through HSCT stages and emotional burden of care for nurses. The central variable would cover all main categories of the study and would make a conceptual relation between them. Figure1 shows the main aspects of the research and their relationship based on the main variable.

**LIMITATIONS**

Although there are some other HSCT centers in Iran, but the information for this study was gathered from the main HSCT center. All other HSCT centers are in contact with this center and get help for starting their activity and nursing education. 

## Discussion

 Findings indicated that supporting patients to go through the HSCT process successfully is the core variable in caring for HSCT patients. A process of care, including 4 main categories and 13 sub-categories happens in a course of vast human interactions. The struggle of patients between life and death is the main concern and driving force for nurses during the patients' care. Two main categories focusing on mortality risk reduction and spiritual development are used by nurses in supportive care for successful transition of HSCT survivors. The outcomes of this supportive care are patients passing the transplant process and the emotional labor of caring. 

Support has been described as a main function of nursing profession. Researchers have had different ideas in describing this support in nursing since support is a complex concept ^       52 ^. Gardner, based on the clinical research on subject of support, describes it as a part of nursing activity with three parameters: physical, social, and emotional^53^. Nurses in the present study performed a variety of care and supportive activities for HSCT patients who had different complications and needs regarding the physical, psychological, social, and financial aspects^54^^,^^55^. So, the emphasis of nursing care was on supportive care for HSCT patients^56^. Since HSCT is a relatively new concept, the studies focusing on these patients are limited, but previous studies have emphasized the rule of supportive care for HSCT patients^57^^,^^58^. Nurses apply the strategies for supporting HSCT patients and try to reduce the risk of death by prevention, monitoring, management of dangers, following-up, continuing education program and providing spiritual care. Nurses did it by counseling, instilling hope and fading out the bitter realities as mentioned in another studies^59^ .By these strategies, caring achievements were emerged^60^.

In studies conducted on cancer patients, the importance of supportive care in the form of giving patients information about changing their way of life and emotional support have been emphasized ^61^. One study from Iran has indicated that “support” is an essential and multidimensional need which should be continuously given to cancer patients to improve their chances of copping with cancer^62^. The support given to patients in the present study was also to help patients to pass through HSCT successfully. In a study of HSCT patients with the aim of understanding how these patients keep courage and pull through this demanding therapy, writing a positive story has been recognized as a central variable. Patients were aware of this concept and made many efforts in order to write a positive story, to keep courage and faith. In this study, the nurses had a central role in helping patients to write this positive story and to pass through the hardest and most despondent moments ^38^.

In another study with interpretative phenomenological approach about nursing of HSCT patients, compassionate presence was identified as the essence of nursing practice in these wards, which showed what the care for these patients is and how this care is done^36^. In another study which has evaluated the caring from the perspective of nurses working with patients in three Canadian acute psychiatric hospital settings using grand theory approach, protective empowering was identified as the main variabl^12^. 

The core variable has been reported in several studies performed in Iran in the care of different patients. In emergency setting, the core variable has been identified as stabilizing the situation, which includes continuous and conscious activities to reduce the pain and suffering among patients and their exact, fast and comprehensive care^63^. In general surgery wards, nurses react to unfavorable workplace environmental factors by limiting their activities and doing only routine tasks^64^. 

It is obvious that the difference in the nature of disease, needs and problems of patients explain the difference in the core variable of these studies. The nurses in the present study provided supportive care for patients struggling between death and life to help them pass through the treatment procedure. 

Identifying and studying the factors affecting the supportive behaviors of nursing staff in caring for HSCT patients in future studies would give us a better view on how to promote the support. The present study indicated the importance of support in successful passage through HSCT. Extracting workable patterns from this theory and testing their usefulness through experimental studies is suggested. Further studies about the educational and supportive priorities of HSCT nursing staff are needed. 

## CONCLUSION

 Supporting patients to undergo HSCT successfully will happen while the patients are fighting for their life, and will continue after HSCT. Nurses tried to help their patients to pass through limbo by providing supportive care services.

Supportive care for successful passage from HSCT might be identified as a guidance for providing care to HSCT patients. Education, emotional support, protection and promoting hope, and the use of coping strategies will increase their chance of success in recovery.

The importance of supportive care in helping HSCT patients should be integrated in the educational material for HSCT nurses. Systematic training of health care providers and families on how to care for these patients before and after HSCT would be a step toward better outcomes. 

Creating social institutions for dealing with HSCT patients, as well as setting up a consultation center for post-HSCT patients and their families would result in better care for these patients.
